# Immunomodulation in the Treatment of Refractory Catastrophic Antiphospholipid Syndrome

**DOI:** 10.1155/2018/1041396

**Published:** 2018-04-01

**Authors:** Karthik Nath, Andrew McCann

**Affiliations:** ^1^Department of Haematology and Bone Marrow Transplantation, Townsville Hospital, Douglas, QLD, Australia; ^2^Department of Vascular Medicine, Princess Alexandra Hospital, Brisbane, QLD, Australia

## Abstract

Catastrophic antiphospholipid syndrome is a rare condition with high morbidity and mortality. We present a refractory case of catastrophic antiphospholipid syndrome with a view to highlight the importance of early identification and aggressive treatment of this condition. A 36-year-old female presented with clinical manifestations of multiorgan vascular occlusion with a known history of primary antiphospholipid syndrome. The presentation was on a background of a recent change of her long-term anticoagulation from warfarin to therapeutic low-molecular-weight heparin. Given that multiorgan involvement with 3 organ systems occurred nearly simultaneously, a diagnosis of probable catastrophic antiphospholipid syndrome was made. Prompt therapeutic anticoagulation, antiplatelet, and glucocorticoid therapy was commenced. Despite this, the patient continued to demonstrate clinical features concerning for ongoing small vessel occlusion necessitating aggressive immunomodulatory therapy in the form of intravenous immunoglobulin, plasma exchange, and rituximab.

## 1. Introduction

Antiphospholipid syndrome (APS) is a multisystem autoimmune disease characterised by vascular thrombosis and/or pregnancy morbidity in the presence of at least one of the circulating antiphospholipid antibodies [1]. Catastrophic APS is a rare, underrecognised and life-threatening variant of APS, occurring in only 1% of persons with APS [2].

In this case report, we aim to highlight the importance of early recognition and an aggressive management strategy that is required in patients presenting with catastrophic APS. We also show the increasing role for immunomodulatory agents in this condition.

## 2. Case Presentation

A 36-year-old Caucasian female with a previous diagnosis of triple antibody-positive primary antiphospholipid syndrome (APS) presented with a 4-day history of left-sided nonpleuritic chest pain. This was on the background of having recently been converted from warfarin (target INR 2.0-3.0) to therapeutic low-molecular-weight heparin (clexane, 80 mg, twice daily) in an attempt to conceive. There was no history of in vitro fertilisation (IVF), and pregnancy testing was negative.

**Figure 1:**
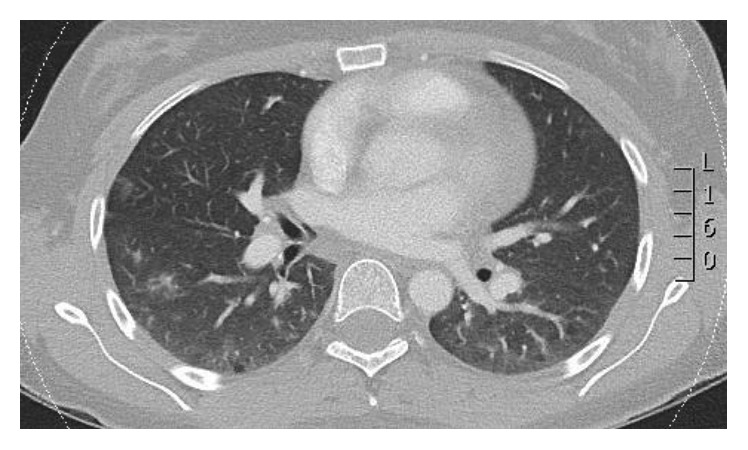
CTPA showing multifocal areas of ground-glass attenuation consistent with pulmonary haemorrhage.

On assessment she was found to have an elevated serum troponin at 6.0 mcg/L and nonevolving ST depression in the lateral leads of her electrocardiogram. Computed tomography pulmonary angiography (CTPA) was negative for pulmonary embolism but showed features of ground-glass opacities in the right peripheral lower lung consistent with pulmonary haemorrhage (Figure 1). Transthoracic echocardiography demonstrated regional wall motion abnormalities suggestive of a possible ischaemic event. The patient was subsequently admitted to the coronary care unit with a diagnosis of a non-ST elevation myocardial infarct on the background of known APS. Pulmonary haemorrhage was presumed secondary to microvascular thrombi within the pulmonary vascular tree.

**Figure 2:**
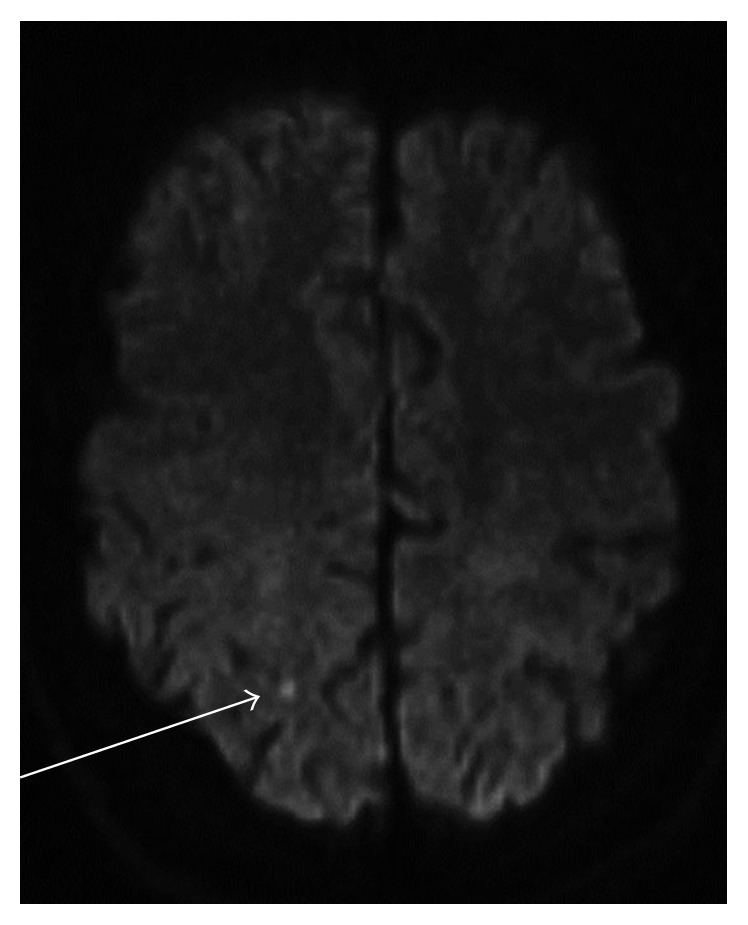
MRI brain demonstrating a focus of restricted diffusion in the right parietal lobe in keeping with acute infarction.

Triple antithrombotic therapy was promptly commenced with intravenous heparin (using anti-Xa monitoring) and dual antiplatelet therapy (aspirin, 100 mg daily, and clopidogrel, 75 mg daily for the cardiac ischaemic event). In addition, atorvastatin (40 mg, daily) and hydroxychloroquine (400 mg, daily) were commenced. Despite adequate anticoagulation and antiplatelet therapy, the patient had recurrent episodes of chest pain. Magnetic resonance imaging (MRI) of the brain also demonstrated an acute right parietal infarct in the setting of new symptoms of transient left hemiparesis (Figure 2).

The involvement of three organ systems within a week was diagnostic of probable “catastrophic” APS. Aggressive immunomodulatory and immunosuppressive therapy was subsequently commenced in addition to the antithrombotic measures. The patient received pulsed methylprednisolone (1 gram/day × 3 doses), intravenous immunoglobulin (0.4 g/kg daily for 5 days), plasma exchange, and rituximab (500 mg/week × 2 doses).

Although the epicaridal coronary arteries appeared normal on coronary angiography, microvascular thrombotic occlusion was thought to be the likely cause of the significant troponin rise with regional wall motion abnormalities. The patient subsequently remained asymptomatic and was discharged on dual antiplatelet therapy and warfarin with an INR target of 3.0-3.5.

## 3. Discussion

The diagnosis of catastrophic APS is made when there is multiple (≥3) organ involvement with vessel occlusion occurring simultaneously or in less than a week in a person with APS [1]. This is usually in the presence of antiphospholipid antibodies in high titre [2]. A definitive diagnosis can only be made by confirmation by histopathology of small vessel occlusion, otherwise cases are defined as probable catastrophic APS [1]. In contrast to APS, where large vessel occlusion occurs, patients with catastrophic APS predominantly develop recurrent vascular occlusions affecting small vessels [3].

Although rare and only occurring in 1% of patients with APS [4], catastrophic APS is considered a life-threatening condition with significant morbidity and mortality. Long-term outcome studies have shown that over 40% of patients died at the time of the initial event, with 26% of survivors developing further thrombotic events [5]. There is often a clear precipitant which includes infection, surgery, anticoagulation withdrawal, malignancy, and change in medication [6]. With this case, we illustrate the importance of exercising caution when considering changes to anticoagulant therapy in persons with APS. Given the close temporal relationship between the patients' presentation and conversion from warfarin to low-molecular-weight heparin, this change in therapy may have contributed to the development of catastrophic APS.

Given the rarity of the condition, there is no standardised treatment algorithm for the management of catastrophic APS (CAPS). However, the mainstay of therapy in CAPS remains aggressive anticoagulation, treatment of any precipitating factors, and early use of corticosteroid therapy. Plasma exchange, rituximab, intravenous immunoglobulin, cyclophosphamide, and terminal complement inhibition should also be considered [6]. The treatment regimen used in this case is supported by recommendations made by the catastrophic antiphospholipid task force report summary. The report recommends consideration of a triple therapy approach (anticoagulation, glucocorticoid, plasma exchange, and/or intravenous immunoglobulin) with the addition of rituximab in the refractory patient [3].

An international database known as the “CAPS registry” provides clinical data on all reported cases and is a useful reference for clinicians [7]. Rituximab, an anti-CD20 monoclonal antibody, had been used in 20 of 441 patients in the CAPS registry as of May 2013 [8]. 75% of these patients recovered from the acute event, and it must be noted that in 12 cases, rituximab was used as a second-line agent. Despite being a small cohort, these findings support the consideration of rituximab in the treatment algorithm for catastrophic APS. Likewise, there are case reports suggesting a role for the terminal complement inhibitor eculizumab in relapsing and refractory cases of catastrophic APS [9].

## 4. Conclusion

This case illustrates the importance of early identification of catastrophic APS, which in itself requires an awareness of potential triggers. An aggressive and multidisciplinary treatment strategy should be implemented without delay, and novel immunomodulatory therapies should be considered, especially in the refractory case.

## References

[1] Erkan D., Espinosa G., Cervera R. (2010). Catastrophic antiphospholipid syndrome: updated diagnostic algorithms. *Autoimmunity Reviews*.

[2] Sciascia S., Lopez-Pedrera C., Roccatello D., Cuadrado M. J. (2012). Catastrophic antiphospholipid syndrome (CAPS). *Best Practice & Research Clinical Rheumatology*.

[3] Cervera R., Rodriguez-Pinto I., Colafrancesco S. (2014). 14th International Congress on antiphospholipid antibodies task force report on catastrophic antiphospholipid syndrome. *Autoimmunity Reviews*.

[4] Cervera R., Bucciarelli S., Plasin M. A. (2009). Catastrophic antiphospholipid syndrome (CAPS): descriptive analysis of a series of 280 patients from the “CAPS Registry”. *Journal of Autoimmunity*.

[5] Erkan D., Asherson R. A., Espinosa G. (2003). Long term outcome of catastrophic antiphospholipid syndrome survivors. *Annals of the Rheumatic Diseases*.

[6] Ortel T. L., Erkan D., Kitchens C. S. (2015). How I treat catastrophic thrombotic syndromes. *Blood*.

[7] CAPS Registry (2017). *European Forum on Antiphospholid Antibodies*.

[8] Berman H., Rodriguez-Pinto I., Cervera R. (2013). Rituximab use in the catastrophic antiphospholipid syndrome: descriptive analysis of the CAPS registry patients receiving rituximab. *Autoimmunity Reviews*.

[9] Wig S., Chan M., Thachil J., Bruce I., Barnes T. (2016). A case of relapsing and refractory catastrophic anti-phospholipid syndrome successfully managed with eculizumab, a complement 5 inhibitor. *Rheumatology*.

